# Ultrasensitive Tunable Terahertz Sensor via Critical Coupling‐Enhanced Fano Resonance

**DOI:** 10.1002/nap2.70111

**Published:** 2026-05-03

**Authors:** Fei Yan, Chenxiang Liu, Yu Wang, Xingdong Liu, Alexander I. Solomonov, Mikhail V. Rybin, Li Li, Hao Tian

**Affiliations:** ^1^ School of Physics Harbin Institute of Technology Harbin China; ^2^ School of Physics and Engineering ITMO University Saint Petersburg Russia; ^3^ Ioffe Institute Saint Petersburg Russia; ^4^ Heilongjiang Provincial Key Laboratory of Advanced Quantum Functional Materials and Sensor Devices Harbin China; ^5^ Key Laboratory of Micro‐Nano Optoelectronic Information System Ministry of Industry and Information Technology Harbin China

**Keywords:** Fano resonance, tunable critical coupling, ultrasensitive THz sensing

## Abstract

High‐performance terahertz (THz) sensing holds significant promise for label‐free medical diagnostics and environmental monitoring, owing to unique fingerprint spectra of biomolecules. However, THz sensing technology still faces limitations, including low sensitivity, low quality factor (*Q*‐factor), restricted figure of merit (FOM), and nontunability. Here, we propose a cavity‐enhanced scheme for an ultrasensitive tunable THz sensor, integrating a graphene‐strip metasurface, a microcavity with dielectric analytes, and a gold reflective layer. We reveal a new physical mechanism allowing tunable critical coupling to maximize light–matter interaction, which involves strong coherent Fano effect arising from dipole localized surface plasmon resonance of graphene‐strip metasurface, coupled with surface plasmon polaritons excited in gold reflective film, and a highly confined cavity mode with narrow absorption linewidth. Hybrid coupling mechanism leads to highly localized field within the microcavity, significantly enhancing light–matter interaction and facilitating THz ultrasensitive sensing. Results show the critical coupling resonance scheme substantially improves the sensitivity and FOM. The sensor demonstrates a high sensitivity of 2.7 THz per refractive index unit (RIU) with a normalized sensitivity of 1.5 RIU^−1^ and a record‐high FOM exceeding 2.54 × 10^6^. The sensing performance is tuned by electrically controlling graphene gate voltage. The proposed scheme is promising for on‐chip integrated THz biochemical sensors.

## Graphical abstract

This work presents a hybrid terahertz sensor via the combination of graphene‐strip metasurface, cavity resonance, and gold film polaritons. The sensor achieves critical coupling with an ultrahigh‐*Q* resonance and tunable FOM with stable high sensitivity via gate voltage regulation, exhibiting excellent performance for on‐chip integrated biochemical sensing.

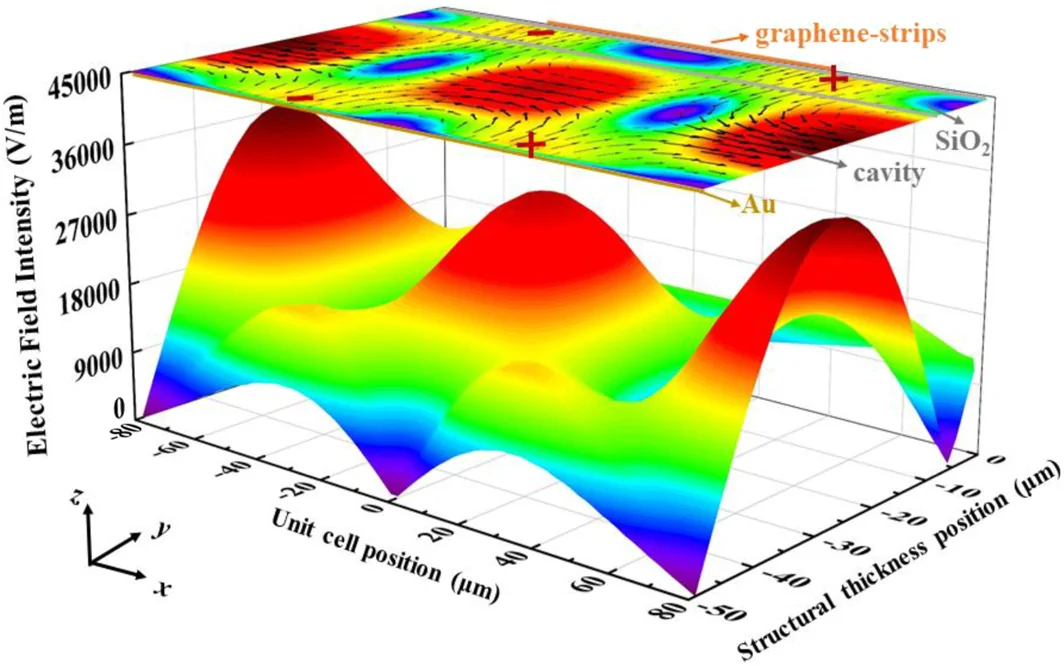

## 1 Introduction

Biochemical sensing enables real‐time medical diagnostics and environmental monitoring, offering distinct advantages such as high sensitivity, compact size, high efficiency, and low power consumption [1, 2, 3, 4]. Terahertz (THz) radiation, which provides valuable information about numerous biomolecules, is one of the most promising platforms for biosensing owing to its unique properties, including low photon energy and deep penetration depth [5, 6, 7, 8, 9]. THz sensing is a novel approach for nondestructive label‐free detection of biochemical molecules with high sensitivity and excellent signal‐to‐noise ratio [10, 11].

The inherent highly localized fields at the subwavelength scale render THz sensing particularly beneficial for miniaturized chip‐scale sensors, which are essential for biomolecule detection in aqueous environments [12, 13, 14, 15, 16, 17]. To evaluate the sensing performance, the figure of merit (FOM), defined as the ratio of sensitivity to resonance linewidth, is widely employed [18]. Various highly sensitive THz biosensors have been presented, both theoretically and experimentally [11, 16, 19, 20, 21, 22, 23, 24, 25, 26]. Ranjan Singh et al. demonstrated a nanofluidic THz metasensor for aqueous analytes, achieving a sensitivity of 93.85 GHz/RIU, although with a FOM below 10 [16]. Yan et al. theoretically presented an ultrasensitive tunable THz sensor using a graphene plasmonic grating, achieving a sensitivity of 4.2 THz/RIU and a normalized sensitivity of 0.45 RIU^−1^, although with a limited FOM of 12.5 [11]. Here, the normalized sensitivity is defined as the ratio of the absolute sensitivity to the resonance frequency, facilitating fair comparison of sensing performance across different spectral regimes [19]. Chen et al. proposed a metamaterial absorber‐integrated microfluidic THz sensor for ethanol and glucose detection [19]_,_ experimentally demonstrating a sensitivity of 0.2 THz/RIU and theoretically attaining 3.5 THz/RIU with normalized sensitivity of 0.55 RIU^−1^, yet with a FOM consistently below 20. Theoretically, Peng et al. introduced a SiO_2_ dielectric diffraction grating‐integrated microcavity sensor for liquid analytes, achieving a normalized sensitivity of 0.53/RIU and an FOM exceeding 600 [26]. However, the significant size mismatch between microscopic biomolecules and macroscopic THz wavelengths remains challenging: it weakens light–matter interactions, severely limiting sensing performance. So far, both theoretical explorations and experimental demonstrations have only attained normalized sensitivity below 1 RIU^−1^, whereas the FOM remains confined to the order of 10^3^. This longstanding limitation prevents further enhancement of sensor capabilities, and innovative solutions are urgently needed for the development of high‐performance THz biochemical sensors. Therefore, exploring novel mechanisms to enhance light–matter interactions is crucial for achieving high‐sensitivity sensing for various applications.

In the paper, we propose a resonant cavity‐coupled scheme for an ultrasensitive tunable THz sensor for the detection of microdose liquid analytes. The integrated platform consists of a graphene‐strip metasurface on a SiO_2_ substrate, a microcavity, and a gold reflective layer. Tunable critical coupling mechanism, employing a hybrid Fano resonance, arises from the coherent interaction between a dipole localized surface plasmon resonance (LSPR) of the graphene‐strip metasurface, surface plasmon polaritons (SPPs) excited in the gold film, and a Fabry–Perot cavity mode. Field confinement inside the microcavity, induced by a sharp Fano resonance, strongly enhances the spatial overlap between the probe field and a liquid analyte. The proposed sensor significantly improves both the sensitivity and FOM in the THz band, achieving a high sensitivity of up to 2.7 THz/RIU with a normalized sensitivity of 1.5 RIU^−1^ and a record FOM exceeding 2.54 × 10^6^. Voltage tuning enables amplitude optimization to improve the signal‐to‐noise ratio, allowing dynamic tuning and enhancing FOM by over two orders of magnitude while maintaining stable high sensitivity. The sensor not only solves the technical problem of stable operation and independent FOM control under interference but also proves to be robust and widely adaptable for biochemical sensing applications, thereby demonstrating significant practical value.

## 2 Model and Materials

Figure 1a illustrates the overall structure of the proposed THz sensor, which consists of a SiO_2_ quartz wafer, a monolayer graphene‐strip metasurface, a sensing cavity, a gold reflective layer, and a substrate. The graphene‐strip metasurface is attached to the quartz wafer, and a 200 nm‐thick gold layer coated on the bottom substrate acts as a mirror. Figure 1b shows the front view and a schematic of the system. The structure features a SiO_2_ slab of thickness *h* covered by a periodic graphene‐strip metasurface with period *p* and strip width *w.* Gold completely reflects the incident field, enhancing the light absorption by the analyte material. A microfluidic channel is formed between the quartz wafer and the substrate for sensing in aqueous environment. In the sensing system, the graphene‐strip metasurface supports an LSPR, the SiO_2_ substrate acts as a dielectric spacer layer, the dielectric microcavity localizes the photon mode, and the gold reflective film is used for SPP excitation. The measured refractive index of SiO_2_ quartz is 2.14 [14]. All geometric parameters were optimized with the iterative optimization method. The period *p* and width *w* were optimized with the *w*/*p* ratio varying from 0.2 to 0.8 as shown in Supporting Information S1: Figure S1b. The selected optimal ratio *w* = 0.5*p* allowed Fano resonance to achieve critical coupling with the narrowest linewidth. The metasurface period is *p* = 160 μm, and the strip width is *w* = *p*/2 = 80 μm. The thickness of the SiO_2_ quartz wafer is *t* = 9 μm, and the microcavity thickness *l* will be varied to find the best conditions. The microcavity can be filled with liquid or gaseous analytes with a specific refractive index, for example, ethanol with an index of 1.6 or glycerol with an index of 1.82 [11, 16, 19]. Unlike conventional designs [11, 12, 13, 14, 15, 16, 17, 18, 19, 20, 21, 22, 23, 24, 25, 26], the proposed Fano critical coupling scheme can enhance the sensitivity of THz absorption through precision design, significantly enhancing the light–matter interaction. The synergistic effects between the LSPR of the graphene‐strip metasurface, the highly confined cavity mode, and the SPPs excited by strong reflection from the gold film are employed to establish strong multimode coherent coupling. This hybrid interaction generates a pronounced coherent Fano effect, highly sensitive to the surrounding environment. This results in a strongly localized electromagnetic field within the microcavity, greatly enhancing the spatial overlap between the resonant modes and liquid analytes. Thereby, our system overcomes the limitations of conventional designs, enabling high‐performance ultrasensitive THz biosensing.

**FIGURE 1:**
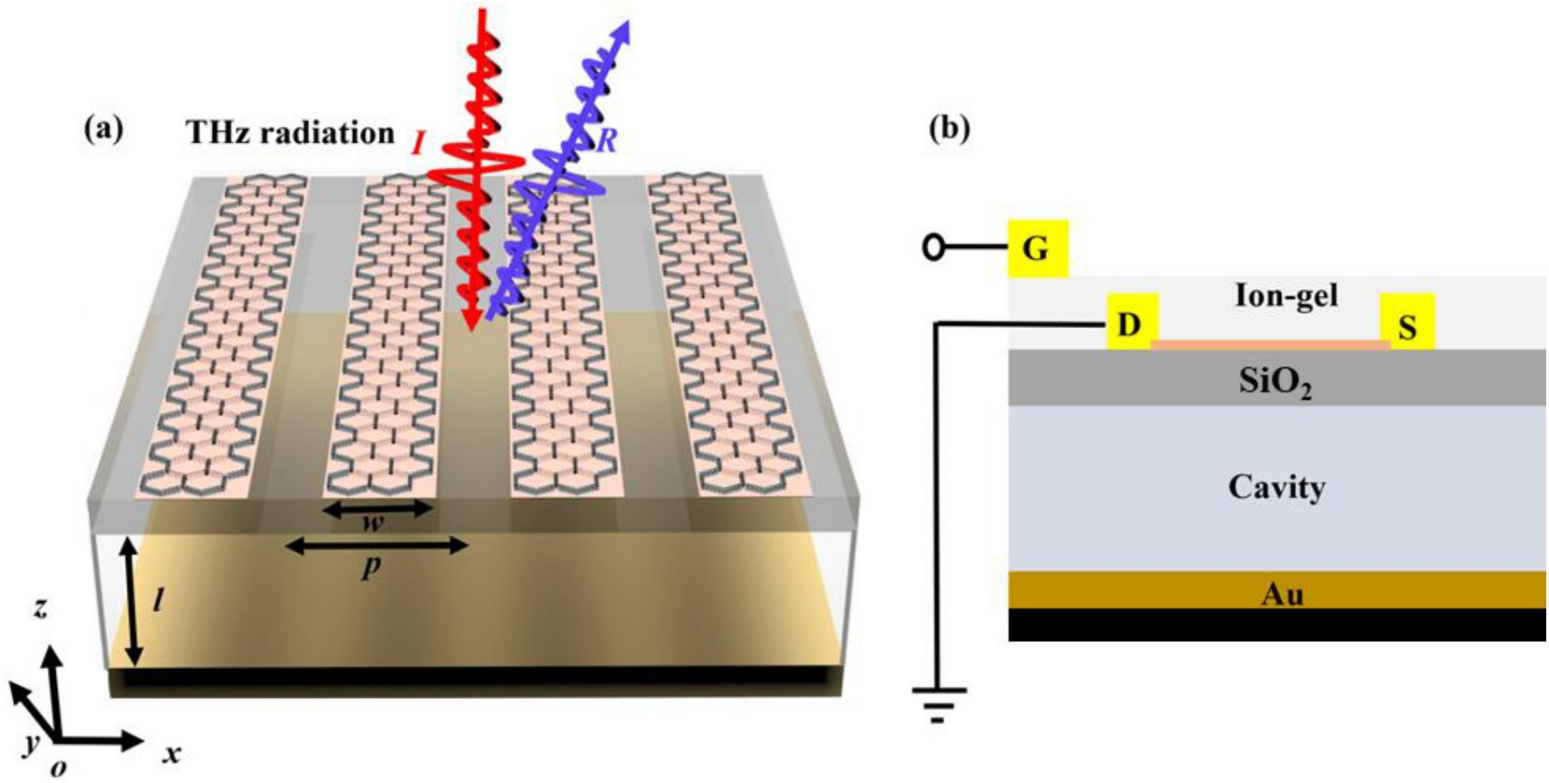
(a) Side view of the proposed utrasensitive tunable THz sensor in aqueous environment. The thickness of the region for liquid analyte sensing is *d*. A gold layer is coated on the substrate. (b) Front view and schematic representation of the system. The proposed structure consists of a SiO_2_ slab of thickness *h* covered by a periodic grating of graphene strips with period *p* and width *w.*

The graphene nanoribbon is periodically patterned along the *x*‐axis and infinitely extends along the *y*‐axis. The optical conductivity σ(ω) of graphene can be modeled using the random‐phase approximation for THz radiation [27], which arises from Kubo formalism, encompassing both inter‐band and intra‐band contributions [28, 29]. For doped graphene, when the basic condition $k_{B}T\ll E_{f}$ is satisfied, the optical conductivity is described by the following equation [29, 30, 31]:

(1) $$\sigma_{g}\left(\omega \right)=\frac{ie^{2}E_{f}}{\pi \hbar^{2}\left(\omega +i\tau^{-1}\right)}+\frac{ie^{2}}{4\pi \hbar }\ln\left[\frac{2\left|E_{f}\right|-\hbar \left(\omega +i\tau^{-1}\right)}{2\left|E_{f}\right|+\hbar \left(\omega +i\tau^{-1}\right)}\right],$$

where *k*_*B*_ is the Boltzmann constant, *T* is the temperature, *E*_*f*_ is the Fermi energy related to carrier concentration, *ħ* is the reduced Planck constant, *ω* is the angular frequency of the incident light, *τ* is the carrier relaxation time, and *e* is the electron charge. The first and second terms of the equation originate from the intra and interband contributions, respectively. The permittivity of monolayer graphene is expressed as follows [11, 32]:

(2) $$\varepsilon_{g}=1+\frac{i\sigma_{g}}{\varepsilon_{0}\omega t}.$$

Critical coupling can be described by coupled mode theory [33, 34], and the reflection coefficient *R* of the structure can be written as follows:

(3) $$R\equiv \frac{\Lambda }{u}=\frac{j\left(\omega -\omega_{0}\right)+\delta -\gamma_{e}}{j\left(\omega -\omega_{0}\right)+\delta +\gamma_{e}},$$

where *u* and *Ʌ* denote the input and output wave amplitudes, respectively. Here, *δ* is the intrinsic loss rate and *γ*_*e*_ is the external leakage rate. Due to zero transmission, the absorption is as follows:

(4) $$Α=1-{\left|R\right|}^{2}=\frac{4\delta \gamma_{e}}{{\left(\omega -\omega_{0}\right)}^{2}+{\left(\delta +\gamma_{e}\right)}^{2}}.$$

From Equations (3) and (4), the absorption properties of the structure are governed by two key parameters: *δ* and *γ*_*e*_. When the external leakage rate *γ*_*e*_ equals the intrinsic loss rate *δ*, satisfying the critical coupling condition, and the system is at resonance (*ω* = *ω*_0_), perfect absorption can be achieved due to zero reflection.

## 3 Results and Discussion

### 3.1 Strong Coherent Fano Interactions at Critical Coupling

The absorption spectra of the graphene‐ and metal‐strip metasurfaces for the microcavity thickness *l* = 40 μm and a refractive index of 2.1 are shown in Figure 2a. The red and purple lines are the absorption spectra of the graphene‐ and metal‐strip metasurfaces (with strip thickness of 200 nm) under the same conditions, respectively. Clearly, the graphene‐strip metasurface provides significantly higher absorption than the metal‐strip one at the critical coupling condition. An asymmetric Fano resonance with perfect absorption and an extremely narrow linewidth is excited at the critical coupling point at the frequency *f*_0_ = 1.852 THz, exhibiting a full width at half maxima (FWHM) of 1 × 10^−3^ THz and providing low noise and high resolution beneficial for biomedical detection. Considering the challenging control of the liquid channel height with sub‐micrometer precision and the potential deformation induced by fluid pressure during operation, we investigated the effect of the microchannel height deviation on the resonance absorption as shown in Supporting Information S1: Figure S2. The proposed sensing scheme maintains robust critical coupling condition for cavity height deviations in the range from approximately −4 μm to +5 μm (at 0.9 absorbance), which can result from fabrication errors and pressure deformation. In addition, polar liquids, such as ethanol and glycerol, usually exhibit high THz dielectric losses associated with the imaginary part *κ* of the refractive index [16, 35, 36]. We should evaluate the possible influence of these losses on the resonance linewidth. As shown in Supporting Information S1: Figure S3, even in the lossy case with a typical large imaginary part (*κ* = 0.14, corresponding to *α* ≈ 110 cm^−1^), the spectral response maintains a satisfactorily narrow FWHM of 27 GHz. Here, we specifically propose a thin microchannel filled with liquid to enable the sensing scheme. For a liquid layer thickness of merely tens of micrometers, dielectric losses weakly impact the sensing performance as discussed in ref. [19]_._ Furthermore, the *Q*‐factor can be calculated as *Q* = 2π*f*_0_ *P*_*S*_/*P*_*L*_ = *f*_0_/FWHM [22], where *P*_*S*_ and *P*_*L*_ are the stored and dissipated energies, respectively. The numerically calculated *Q‐*factor reaches 1.852 × 10^3^, accompanied by a large modulation depth [(*A*_max_−*A*_min_)/(*A*_max_ + *A*_min_) × 100%] of 90%. Using theoretical calculations with the coupled mode theory, we found the fitting parameters to be *δ* = *γ*_*e*_ = 0.22 × 10^−3^ THz at the frequency *f*_1_ = 1.852 THz. Correspondingly, the *Q*‐factors associated with external leakage and intrinsic loss are defined as *Q*_*δ*_ = *ω*_0_/2*δ* and *Q*_*γe*_ = *ω*_0_/2*γ*_*e*_, respectively. The total quality factor *Q* is estimated by the coupled mode theory using the formula *Q* = *Q*_*δ*_ *Q*_*γe*_/(*Q*_*γe*_ + *Q*_*δ*_), yielding 1.853 × 10^3^. This theoretical value agrees well with the numerical simulation result 1.852 × 10^3^ for the Fano resonance mode. Note that in real THz spectroscopy systems, the incident light typically has a certain divergence angle (i.e., focused beam). The effect of the incident angle on the absorption spectra is shown in Supporting Information S1: Figure S4 of the Supporting Information. The results indicate that the high‐*Q* mode achieved via critical coupling and Fano resonance is quite robust under oblique incidence at a large angle of 30°.

**FIGURE 2:**
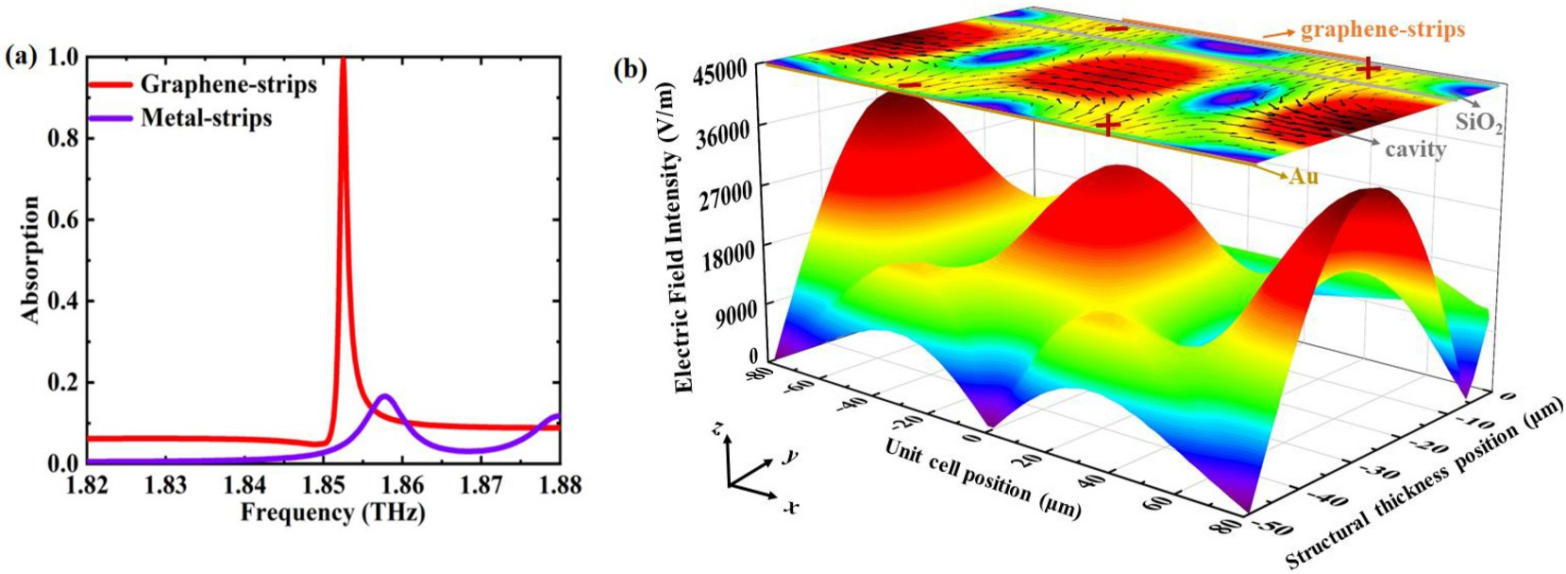
(a) Absorption spectra of the hybrid system when the microcavity with thickness *l* = 40 μm is filled with a material with a refractive index of 2.1 under normal incidence. (b) 3D distribution of the electric field intensity | ***E*** | at the resonance frequency and its projection on the *x*‐*z* plane, where the *z*‐axis represents the electric field intensity. The arrows indicate the field direction.

To illustrate the critical coupling effect, Figure 2b shows the 3D distribution of the electric field intensity |*E*| at the resonance frequency and its projection on the *x*‐*z* plane, with black arrows indicating the electric field direction. Clearly, at *f* = 1.852 THz, light penetrates inside the structure with negligible reflection. The white line in the middle shows a graphene strip. We can see that the electric field is simultaneously localized at the edges of the graphene‐strip metasurface and at the interface with the gold reflective film, while within the microcavity, standing waves form with two antinodes and three nodes. This phenomenon demonstrates that the periodic arrangement of the graphene‐strip metasurface satisfies the Bragg condition, enabling wavevector matching between SPPs in gold and LSPR in metasurface [37, 38]. Opposite positive and negative charges are generated on the surfaces of the graphene‐strip metasurface and the gold reflective film, respectively. The electric field strength across the graphene‐strip metasurface exhibits a characteristic distribution with maxima at both edges and a minimum in the center, demonstrating the successful excitation of dipole LSPR in the graphene‐strip metasurface [39, 40, 41, 42]. The gold reflective film excites SPPs, which can significantly enhance the light absorption and effectively confine the electromagnetic energy. The SPPs excited in the gold reflective film can overcome the diffraction limit of conventional optics, confining electromagnetic field energy within subwavelength‐scale regions. This localization significantly enhances light–matter interactions and dramatically improves the sensing sensitivity. When incident light resonantly couples with free electrons on the gold film surface, it generates locally enhanced electromagnetic hot spots that lead to anomalous perfect absorption in the near‐field region. Optical impedance matching governs this process, as demonstrated in Supporting Information S1: Figure S5. In this system, the graphene‐strip metasurface generates dipole LSPRs, acting as discrete states, and the gold reflective film supports propagating SPPs, acting as continuous states [43]. This distinctive resonance manifests spectroscopically as asymmetric line shapes in the absorption spectrum, which can be quantitatively described by the Fano formula [44]:

(5) $$A\left(E\right)\propto \frac{{\left(q+\Omega \right)}^{2}}{1+\Omega^{2}},$$

where *Ω* = 2(*E*−*E*_0_)/*Γ*; *E* is the energy of incident light, *E*_0_ is the resonance energy, *Γ* is the resonance absorption linewidth, and *q* is the Fano parameter that determines the spectral line shape. According to Equation (5) for the Fano resonance profile, the fitting curve is shown as the blue curve in Figure 3a, with the main fitting parameters *q* = −0.1756 and the resonance linewidth *Γ* = 1.1 × 10^−3^ THz. As Figure 3a shows, when approaching the resonant condition (*ω*→*ω*_0_), the theoretical Fano curve is in excellent agreement with the numerically simulated absorption spectra. Perfect absorption with zero reflection can be explained by impedance matching [45]. Critical coupling can be tuned by adjusting the gate voltage. Figure 3b illustrates variations of absorption intensity and resonance linewidth with gate voltage. As the gate voltage increases, the absorption peak intensity exhibits nonlinear behavior, first increasing and then decreasing, with critical coupling achieved at 50.1 V (indicated by the black vertical line). Meanwhile, the absorption bandwidth is gradually expanding. The reason is, before reaching the critical point, coupling efficiency optimization dominates, enhancing the absorption. Throughout the process, free‐carrier absorption loss gradually increases, continuously reducing the *Q*‐factor and broadening the bandwidth. Beyond the critical point, excessive free‐carrier absorption loss drives the system into an overcoupled state, not only further broadening the resonance linewidth but also reducing the peak intensity [46, 47, 48, 49]. Additionally, from a sensor perspective, there is an optimal range of “coupling strength” achievable through geometry modification and gate voltage adjustment, which can provide the desired balance between high absorption (for a strong signal) and narrow linewidth (for high resolution). As shown in Supporting Information S1: Figure S6, to ensure both strong absorption (≥ 0.95) and narrow linewidth (around 1′10^−3^ THz), the geometric arrangement of the graphene strip arrays should be modified within the range of *w*/*p* = 0.45–0.55 around the critical coupling condition. Meanwhile, the gate voltage can be adjusted within |*V*_*g*_| = 20–100 V. We should note that practical applied gate voltages may reach 45–105 V, considering the high breakdown field of graphene achieved through optimized sample preparation, as suggested in Refs. [50, 51], which guarantees structural integrity without damaging the graphene. In Figure 3c, we further quantitatively calculated the Fano resonance linewidths as a function of Fermi energy *E*_*F*_ in the range of 0.13–0.83 eV under gate voltage control, revealing a linear relationship of *Δ*_*f*_ = 2.14 × 10^−3^ *E*_*F*_ + 1.1 × 10^−4^, with excellent linearity of *R* ^2^ = 0.991.

**FIGURE 3:**
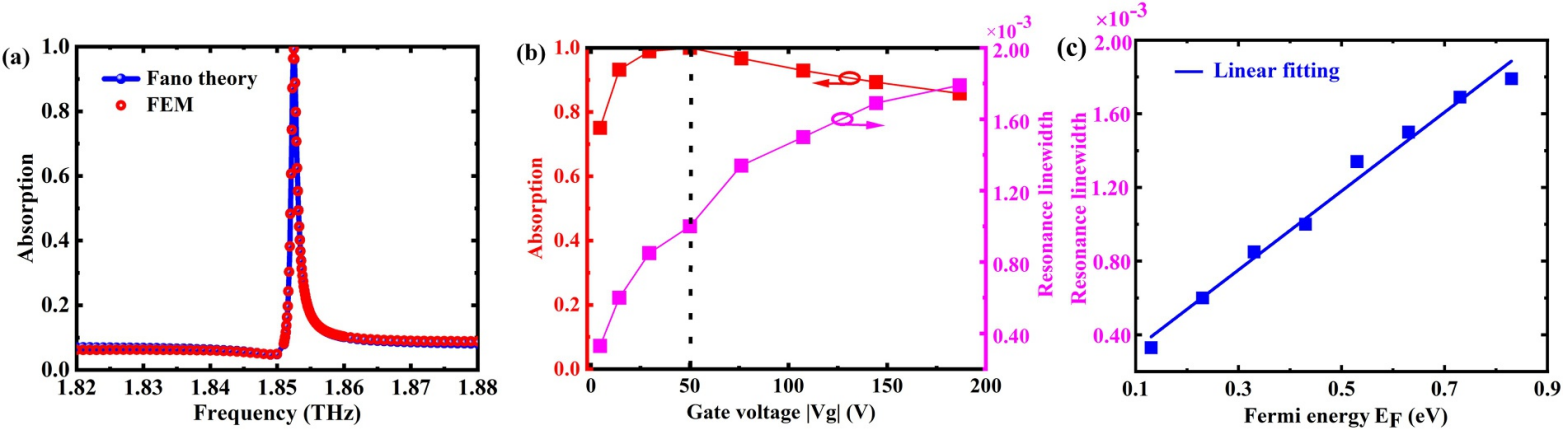
(a) Absorption spectra calculated by FEM simulations and normalized Fano profile theory. (b) Absorption peak intensity and absorption bandwidth versus the gate voltage. (c) Linear relationship between resonance linewidth and Fermi energy.

### 3.2 Ultrasensitive THz Sensing

The absorption bandwidth plays a crucial role in ultrasensitive THz sensing for practical applications. The pronounced field overlap between the excited standing wave and an analyte significantly enhances light–matter interaction at the subwavelength scale, offering an innovative solution for label‐free refractive index detection in environmental monitoring. High *Q*‐factor and low‐loss resonant properties make our system highly promising for biochemical sensing. Generally, sensing performance is characterized by two key parameters: sensing sensitivity *S* and FOM, which are defined as follows [11, 16]:

(6) $$S=\frac{\Delta f}{\Delta n_{1}},$$

(7) $$\text{FOM}=\frac{S}{\text{FWHM}},$$

where Δ*f* is the resonance frequency shift in presence of an analyte and Δ*n*_1_ is the change of the environment refractive index due to the analyte.

As shown in Figure 4a, when the microcavity is filled with dielectric analytes with refractive indices of 1.4, 1.5, 1.6, 1.7, and 1.8, respectively, the resonance spectra are significantly redshifted, whereas the absorption spectra are initially enhanced, and then attenuated. The results show that the excited Fano resonance frequency shifts from 3.03 THz to 1.94 THz, with the remarkable frequency shift of 1.09 THz originating from the highly sensitive response of the high‐*Q* microcavity to refractive index variations; this frequency shift is 47 times higher than the resonance linewidth at *n*_*c*_ = 1.4. As the refractive index of the analyte increases, the effective optical path length *L* = *n*_*c*_ *l* also increases, shifting the standing‐wave condition toward longer wavelengths; consequently, the resonance frequency redshifts [52]. Simultaneously, for a higher refractive index of the analyte, the effective refractive index *n*_eff_ of the electromagnetic modes within the microcavity grows. According to the dispersion relation *ω* = *c* ***k***/*n*_eff_, for a given wavevector ***k***, the resonance frequency *ω* decreases proportionally [53]. Figure 4b illustrates an excellent linear relationship between the resonance frequency and the analyte refractive index, with the fitting coefficient *R* ^2^ approaching 0.985. The high sensitivity of the Fano resonance can reach approximately 2.7 THz/RIU, enabled by key physical mechanisms: strong field enhancement at the gold‐dielectric interface, the sharp asymmetric line shape of the Fano resonance, and effective modulation of the cavity mode by the dielectric environment. This exceptional sensitivity demonstrates that refractive index can be measured with high precision by tracking resonance peak shifts. Figure 4c illustrates the corresponding variations of the *Q*‐factor and the FWHM. The changes in the resonance linewidth are determined by the associated change in damping loss due to the increase in the analyte refractive index [50]. The FWHM values are 2.34 × 10^−2^ THz, 5.50 × 10^−3^ THz, 9.80 × 10^−4^ THz, 8.35 × 10^−5^ and 7.05 × 10^−6^ THz, respectively. Here, we should note that the resonance linewidth can reach the order of 10^−6^ THz. The spectral resolution of current mainstream THz time‐domain spectroscopy (THz–TDS) systems is typically at the GHz level, insufficient for measurement of such narrow spectral features. Besides THz–TDS systems, advanced frequency‐domain continuous‐wave THz spectroscopy systems based on photomixing techniques [54, 55], such as the TOPTICA TeraScan series, can achieve frequency resolution down to 1 MHz (TeraScan 1550) or even single Hz (TeraScan ultra). This may allow the characterization of a MHz linewidth under the existing experimental conditions. The corresponding *Q*‐factors are 1.30 × 10^2^, 4.88 × 10^2^, 1.30 × 10^2^, 2.53 × 10^3^, and 2.75 × 10^5^. Interestingly, both the FWHM and *Q*‐factor exhibit pronounced exponential dependence on the analyte refractive index, with *R* ^2^ = 1, respectively. This phenomenon primarily stems from two physical mechanisms: enhanced optical field localization with compression of the mode volume *V*_mode_, and exponential suppression of radiative losses *γ*_rad_ [56]. Figure 4d illustrates the corresponding changes in the FOM. As the analyte refractive indices *n*_*c*_ range from 1.4 to 1.8, the corresponding FOM values are 1.15 × 10^2^, 4.91 × 10^2^, 2.76 × 10^3^, 3.23 × 10^4^, and 3.83 × 10^5^, respectively. Thus, the FOM linearly grows with the analyte refractive index. Remarkably, when the analyte refractive index falls within the range of 1.6–1.8, the FOM can exceed 10^3^ and even reach up to 10^5^, significantly enhancing the THz sensing performance compared to previous theoretical results [11, 16, 19, 26]. The exceptionally high FOM primarily arises from the strong coherent hybrid coupling mechanism among the graphene dipole LSPR, gold‐film SPPs, and cavity mode. We should note that the geometry of the proposed sensor was optimized specifically for analytes, such as ethanol, liquid paraffin, and gasoline, with refractive indices in the range of 1.4–1.8 [10, 11, 19]. However, for analytes with extremely low refractive indices (e.g., gases with refractive index close to 1), Figure 4b,d indicate that the sensitivity and FOM are compromised, because the critical coupling condition is severely disrupted by the significant refractive index mismatch. To use the hybrid coupling mechanism under low refractive index conditions, the geometry parameters should be optimized for these indices to achieve superior sensing performance. Figure 5a,b show the distribution of the electric field strength | ***E*** | for analyte refractive indices of 1.4 and 1.8, respectively. As the analyte refractive index increases, the electromagnetic field strength is significantly enhanced. Specifically, when the analyte refractive index increases from 1.4 by Δ*n* = 0.4, the field enhancement factor increases at least 14‐fold, significantly improving the electromagnetic field localization and enhancing the light–matter interaction.

**FIGURE 4:**
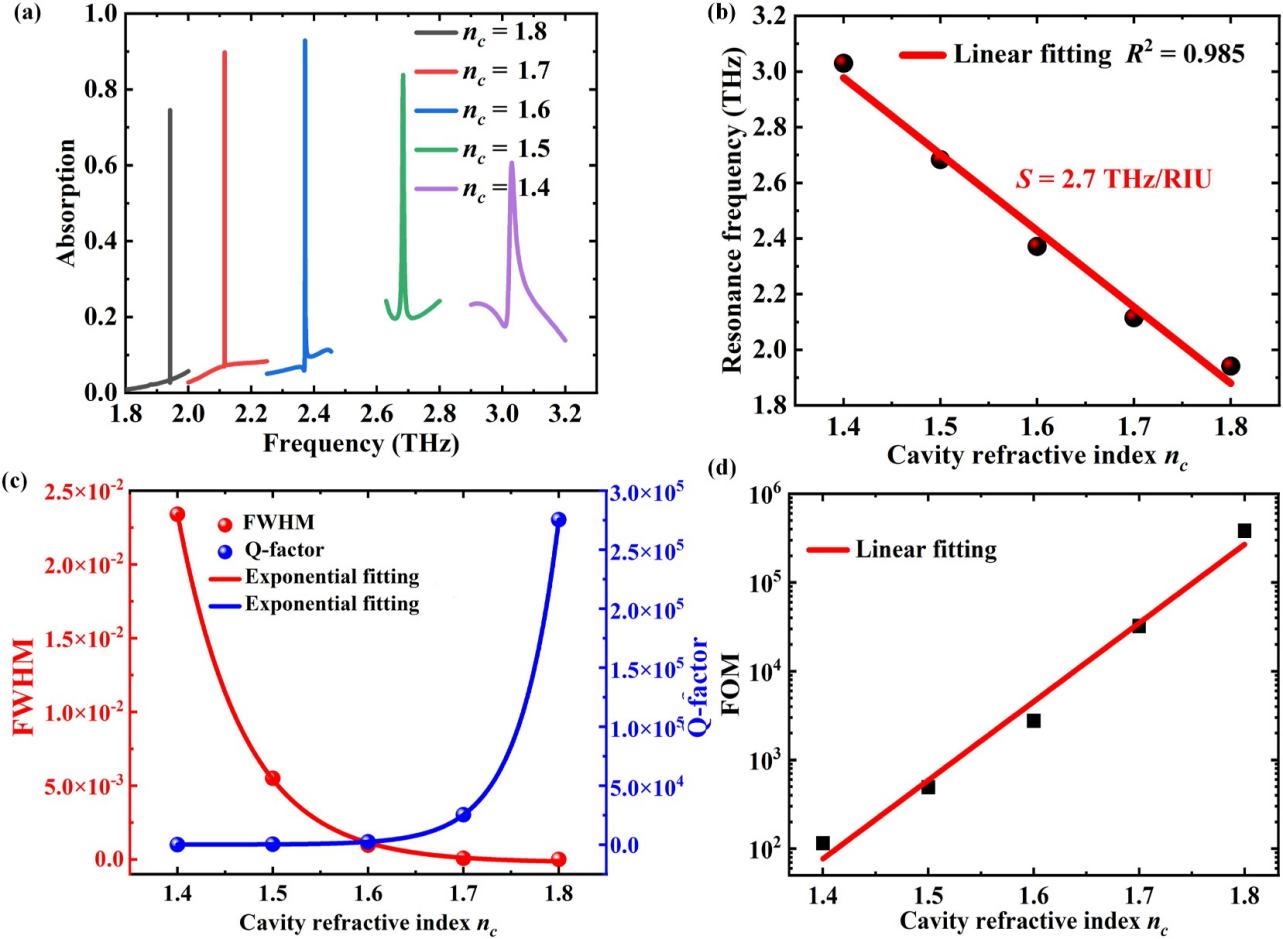
(a) Absorption spectra of the proposed THz sensor filled with analytes with different refractive indices under normal incidence of light. (b) Linear variation of the resonance frequency of the Fano resonance versus the refractive index of the analyte. (c) *Q*‐factor and the absorption FWHM versus the refractive index. (d) FOM versus the refractive index.

**FIGURE 5:**
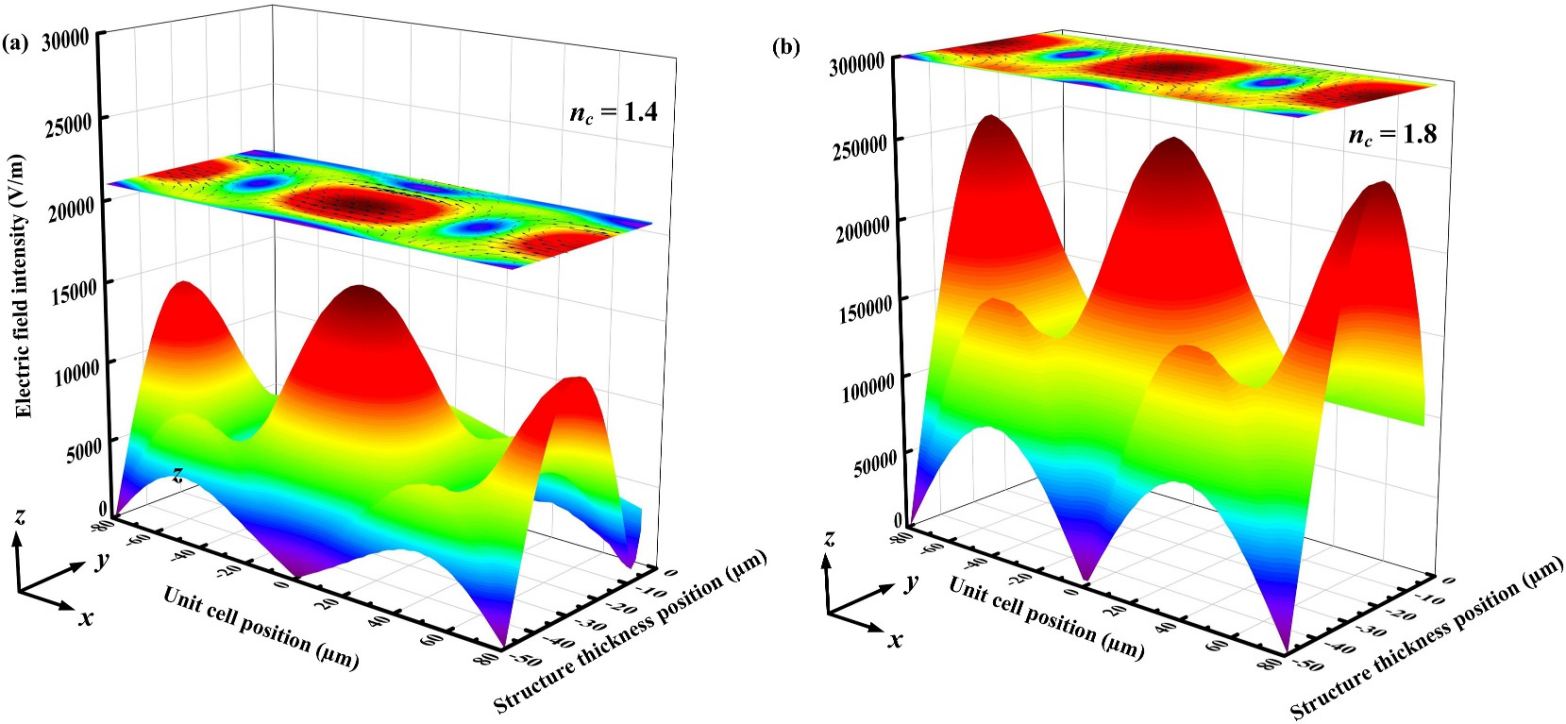
(a) Distribution of the electric field strength |*E*| for an analyte refractive index of 1.4. (b) Distribution of the electric field strength |*E*| for an analyte refractive index of 1.8.

Owing to the electric field of the standing wave formed within the microcavity, the cavity resonance has a particularly significant modulation effect on the Fano resonance. Among the key parameters, the microcavity thickness *l* mainly determines the spatial distribution of the standing‐wave field, critically influencing the hybrid mode. For an analyte refractive index of 1.8, the evolution of resonance frequencies and absorption for various values of cavity thickness *l* are shown in Figure 6a. Within the 34–47 μm range, the resonance frequency decreases linearly with cavity thickness. In the microcavity, the resonance frequency *f* and cavity thickness *l* approximately follow the relation *f* ∝ *c*/(2_*n*eff_ *l*) [43, 52]. This stage is likely dominated by the cavity mode, where the frequency response is solely tuned by the cavity thickness, exhibiting a linear trend. As the thickness increases further, the modal coupling gradually weakens. Due to the different slopes of the dispersion curves of graphene LSPR, gold‐film SPPs, and cavity resonance, the frequency‐cavity thickness relationship deviates from a linear one, gradually declining until the mode degeneracy is lifted, resulting in resonance peak splitting. As the cavity thickness increases from 34 to 58 μm, the absorption intensity first strengthens and then weakens, reaching perfect absorption when the cavity thickness is 45 μm. This originates from the critical coupling condition; at this point, the radiation losses within the cavity and the material absorption loss are balanced, leading to complete energy dissipation [57]. As the cavity thickness increases further, mode mismatch or loss imbalance reduces the absorption. In the gray shaded region (39–47 μm) in Figure 6a, the resonance frequency changes linearly with thickness and the corresponding absorption intensities exceed 0.5, demonstrating excellent absorption performance. When the cavity thickness *l* increases from 39 to 47 μm, the resonance frequencies range from 1.945 THz to 1.917 THz. Notably, during this process, the linewidth of the absorption spectra initially decrease and then increase, with the corresponding linewidths of 4.25 × 10^−5^ THz, 1 × 10^−5^, 0.21 × 10^−5^ THz, 3.3 × 10^−5^, 1 × 10^−4^ THz, 2.3 × 10^−4^, 3.8 × 10^−4^ THz, 6.8 × 10^−4^ THz, and 8.5 × 10^−4^ THz.

**FIGURE 6:**
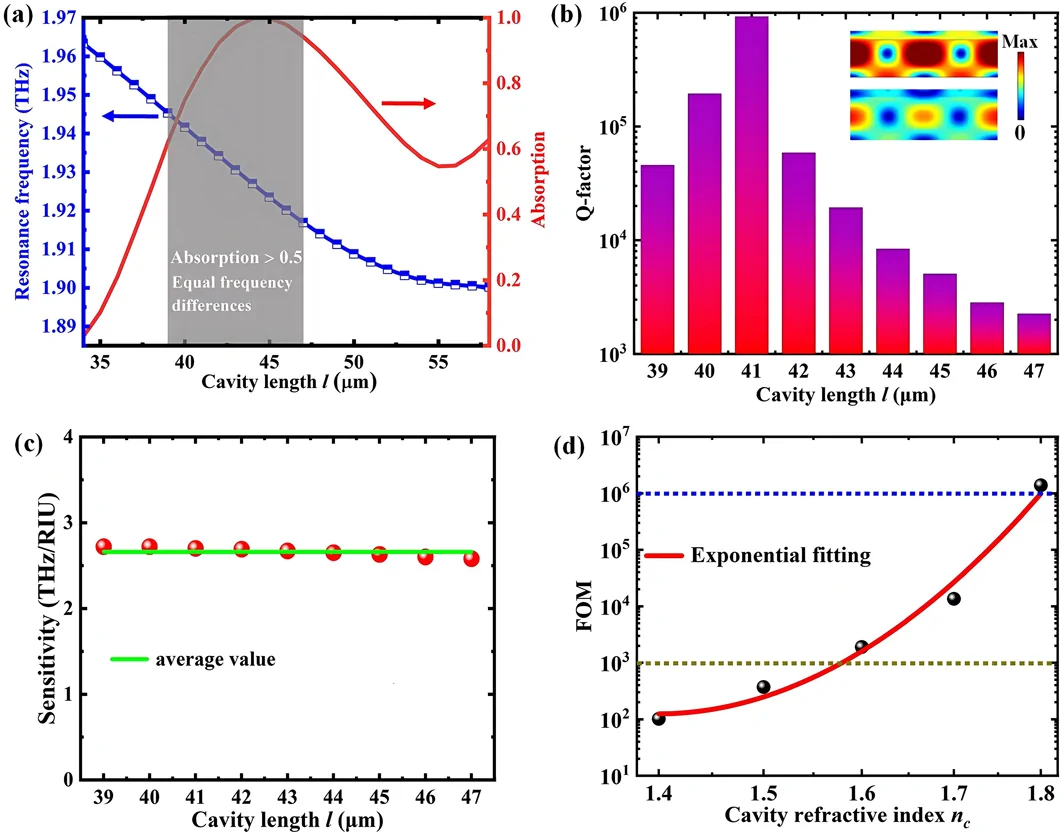
(a) Absorption and resonance frequency as a function of the microcavity thickness *l*. In the gray shaded region (where the microcavity thickness ranges from 39 to 47 μm), the resonance frequency changes linearly with thickness and the absorption intensity exceeds 0.5. (b) Corresponding *Q*‐factor as a function of the microcavity thickness *l*; the insets show the electric field distribution at the resonance frequency for microcavity thicknesses of 41 and 47 μm, respectively. (c) Sensitivity versus the microcavity thickness for an analyte refractive index of 1.8. (d) FOM variations versus the analyte refractive index *n*_*c*_ for a microcavity thickness *l* of 41 μm.

Figure 6b illustrates the evolution of the *Q*‐factor with the microcavity thickness *l*. For the thicknesses of 39–47 μm, the *Q*‐factor exhibits a nonmonotonic trend of initial increase followed by decrease, with specific values of 4.58 × 10^4^, 1.94 × 10^5^, 9.23 × 10^5^, 5.86 × 10^4^, 1.93 × 10^4^, 8.38 × 10^3^, 5.06 × 10^3^, 2.82 × 10^3^, and 2.26 × 10^3^, respectively. This behavior closely correlates with variations in linewidth. Notably, when the cavity thickness is 41 μm, the *Q*‐factor reaches an exceptionally high value of ∼10^6^. The insets provide electromagnetic field distributions for cavity thicknesses of 41 and 47 μm, where the color gradient represents the normalized electric field intensity. At a thickness of 41 μm, the electric field is significantly localized. Near the gold film surface and in the microcavity region, the field is strongly enhanced, and it is moderately localized near the graphene edges. This indicates that the system is in a triple strong‐coupling state involving the graphene LSPR, cavity‐mode resonance, and gold‐film SPPs, maximizing the *Q*‐factor. In contrast, when the cavity thickness increases to 47 μm, the field becomes weakly localized in all regions and the field enhancement almost disappears near the graphene edges. This field distribution suggests a substantial reduction in the coupling strength between the resonance modes, increasing the energy dissipation and, consequently, reducing the *Q*‐factor. Figure 6c presents the sensing sensitivity as a function of the analyte refractive index from 1.4 to 1.8 for different cavity thicknesses *l*. The results demonstrate that the sensing sensitivity remains remarkably stable, averaging approximately 2.7 THz/RIU across refractive index variations, virtually independent of changes in the cavity thickness. This thickness‐independent sensitivity is explained by the relationship between the resonant frequency difference Δ*f* and the thickness variation Δ*l* [41]: they are proportional, that is, Δ*f*/Δ*l* remains invariant. This scaling ensures that the sensing sensitivity Δ*f*/Δ*n*_*c*_ is unaffected by cavity thickness variations. These interesting results demonstrate that through rational design of hybrid resonance systems, a high‐performance sensor robust to geometric parameter variations can be achieved. The FOM evolution with the analyte refractive index is shown in Figure 6d. The results reveal that as the refractive index increases from 1.4 to 1.8, the FOM exponentially grows, with exact values of 1.00 × 10^2^, 3.72 × 10^2^, 1.90 × 10^3^, 1.35 × 10^4^, and ultimately reaches 1.38 × 10^6^. Note that cavity thickness deviation can influence the FOM as discussed in Supporting Information S1: Figure S7 When the cavity thickness deviates by ± 2 μm, the FOM decreases to the order of 10^5^. For deviations of up to ± 4 μm, the FOM remains at a satisfactory level of 10^4^. Through gate voltage tuning, the critical coupling resonance scheme can improve the signal‐to‐noise ratio. This approach further enables dynamic tuning and FOM enhancement by over two orders of magnitude while maintaining stable high sensitivity. More details are shown in Supporting Information S1: Figure S8 of the Supporting Information. We should note that the resonance frequency is dominated by the geometry of the sensing cavity and is directly associated with the equivalent optical path (i.e., the product of the geometric distance and refractive index) [58]. Voltage modulation affects the carrier concentration and damping loss of graphene rather than the optical path of the sensing cavity, thus keeping the resonance frequency insensitive to gate voltage variations. Note that the graphene relaxation time *τ* determines the ohmic loss *σ*′ ∝ 1/*τ* in the THz regime as described by the Kubo formula (Equation 1). From Equation (2), *ε*_*g*_ = 1 + *iσ*_*g*_/*ε*_0_ *ω*, the imaginary part *ε*_*g*_′′ = *σ*′/(*ε*_0_ *ω*) quantifies the absorption loss [31, 59]. Therefore, an increase in the graphene relaxation time *τ* reduces the imaginary part *ε*_*g*_′′ and the resonance linewidth, and improves the FOM. The sensitivity remains stable, being primarily determined by the cavity geometry rather than the graphene damping. Compared with previous state‐of‐the‐art‐results [11, 19, 20, 21, 22, 23, 24, 25, 26, 60, 61, 62, 63] as summarized in Supporting Information S1: Table S1 of the Supporting Information, our scheme demonstrates refractive index sensing with a high sensitivity and outstanding FOM. The proposed critical coupling resonance scheme achieves a high sensitivity of 2.7 THz/RIU and a normalized sensitivity of ∼1.5/RIU, which is 3.5 times higher than that of graphene subwavelength gratings [11]. Particularly notable is the record‐breaking FOM of 2.54 × 10^6^, surpassing those of dielectric metasurfaces by two orders of magnitude [57]—a comprehensive breakthrough in sensing performance. The proposed sensor structure is promising for on‐chip integrated THz biochemical sensors, creating a solid foundation for progress in this field [64, 65].

## 4 Conclusions

We have presented a high‐performance ultrasensitive THz refractive index sensor integrating a graphene‐strip metasurface, a microcavity, and a gold reflective layer for label‐free biochemical sensing. Using a strong coherent Fano resonance, the system enables critical coupling between a dipole LSPR generated by a graphene‐strip metasurface, SPPs excited in the gold reflective film, and a highly confined cavity mode. This unique hybrid coupling mechanism creates strongly localized electromagnetic fields within the microcavity, significantly enhancing light–matter interactions. Due to the high spatial overlap between the probing field and analytes, high‐performance THz sensing is enabled. The high sensitivity reaches up to 2.7 THz/RIU with a normalized sensitivity of 1.5 RIU^−1^, and a record FOM exceeds 2.54 × 10^6^. Notably, the proposed sensing scheme demonstrates excellent robustness. When the gate voltage of the graphene strips is electrically tuned, the resonance frequency does not change, and the sensitivity remains stable, whereas the FOM can be tailored to meet the requirements of different applications. The proposed critical coupling resonance scheme is well‐suited for the development of miniaturized ultrasensitive THz biochemical sensors.

## Author Contributions

**Fei Yan:** writing – original draft, investigation, funding acquisition. **Chenxiang Liu:** data curation, methodology. **Yu Wang:** formal analysis. **Xingdong Liu:** data curation. **Alexander I. Solomonov:** validation. **Mikhail V. Rybin:** writing – review and editing, software. **Li Li:** writing – review and editing, supervision, funding acquisition. **Hao Tian:** writing – review and editing, supervision, funding acquisition, project administration.

## Conflicts of Interest

The authors declare no conflicts of interest.

## Supporting information


Supporting Information S1

## Data Availability

The data that support the findings of this study are available from the corresponding author upon reasonable request.
