# Supplementary material for: Ultrasensitive Tunable Terahertz Sensor via Critical Coupling‐Enhanced Fano Resonance
Source: Nanophotonics. 2026 May 3;15(9):e70111. doi: 10.1002/nap2.70111 (PMC13642091; doi:10.1002/nap2.70111)
Supplement: Supplementary file 1 [file NAP2-15-e70111-s001.pdf]

Supporting Information

Ultrasensitive Tunable Terahertz Sensor via Critical Coupling-Enhanced Fano Resonance

Fei Yan[1], Chenxiang Liu[1], Yu Wang[1], Xingdong Liu[1], Alexander I. Solomonov[1,2], Mikhail V. Rybin[1,2,3,*] , Li Li[1,*], and Hao Tian[1,4,5,*]

[1]*School of physics, Harbin Institute of Technology, Harbin 150080, China*
[2]*School of Physics and Engineering, ITMO University, St. Petersburg 197101, Russia*
[3]*Ioffe Institute, St. Petersburg 194021, Russia*
[4]*Heilongjiang Provincial Key Laboratory of Advanced Quantum Functional Materials and Sensor devices, Harbin 150001, China*
[5]*Key Laboratory of Micro-Nano Optoelectronic Information System, Ministry of Industry and Information Technology, Harbin 150001, China*
[*]*Corresponding email*: *lili.phys@hit.edu.cn*; *tianhao@hit.edu.cn*; *m.rybin@mail.ioffe.ru*

The THz optical response can be controlled via critical coupling by adjusting the filling fraction w/p associated with the ratio of graphene-strips width and the metasurface period. Fig. S1(a) demonstrates the evolution of absorption spectra as a function of the graphene-strip width. The results indicate that the resonant frequency of thea hybride LSPR-SPP mode remains essentially constant regardless of width variations, whereas both the absorption intensity and spectral lineshape exbihit significant width-dependent modulation. Fig. S1(b) shows the optimization process of period p and width w of the graphene-strips, where the w/p ratio is scanned from 0.2 to 0.8. The optimal ratio of $w = 0.5p$ is chosen to ahcieve critical coupling of the Fano resonance simultaneously with the narrowest linewidth.

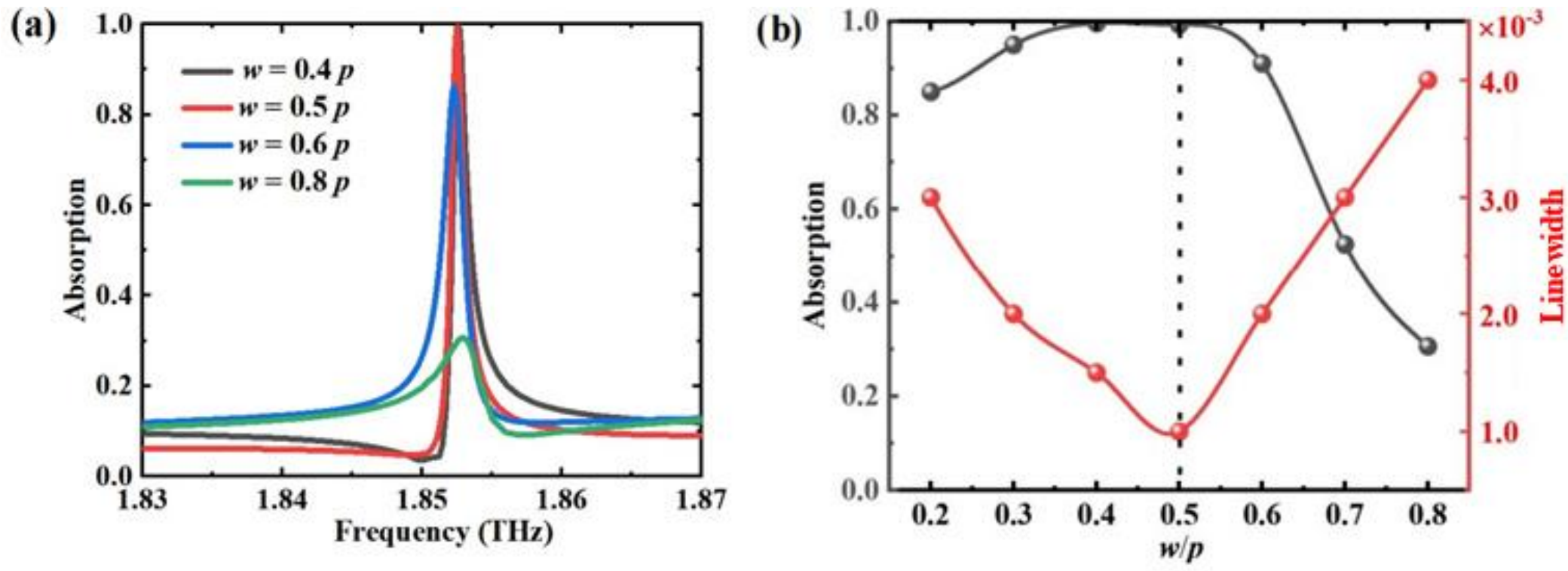

Figure S1. (a) Evolution of the absorption spectra with varying graphene-strips width. (b) Variation of the absorption and linewidth as a function of the width-to-period ratio w/p.

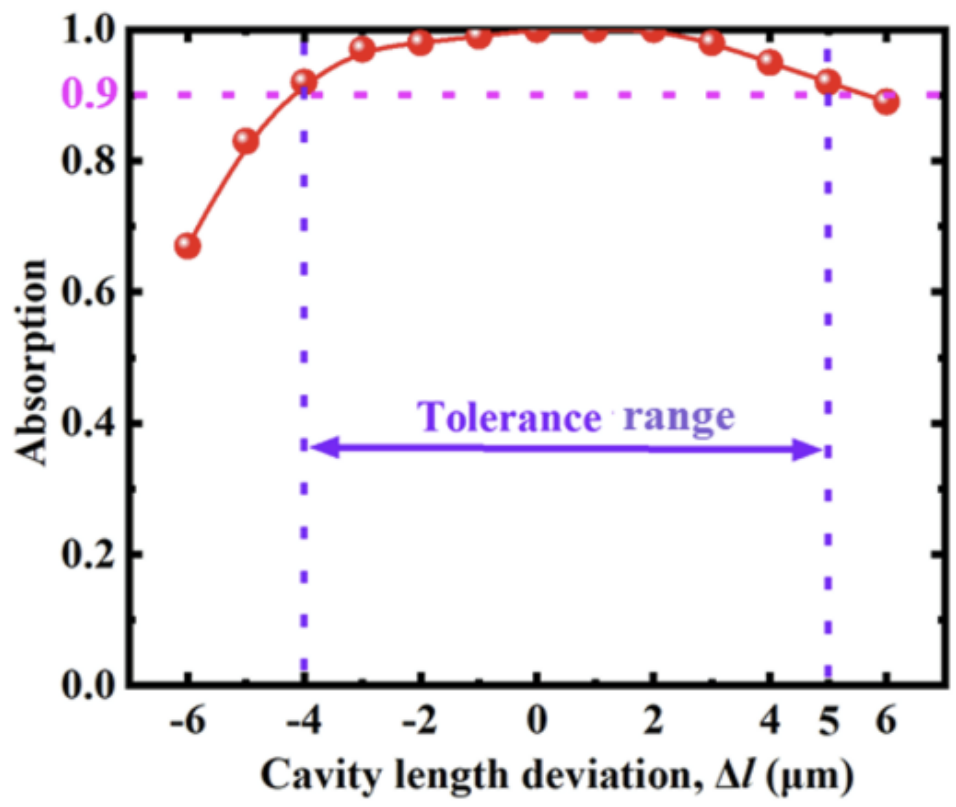

Figure S2. Fabrication tolerance analysis of the critical coupling condition.

The absorption spectra are plotted as a function of cavity length deviation Δl, as shown in Figure S2. A horizontal dashed line marks an absorption level of 0.9, defining the acceptable performance. The proposed sensing scheme maintains robust critical coupling condition within a geometry tolerance range of approximately −4 μm to +5 μm for cavity length, demonstrating resilience to fabrication errors or pressure deformation.

Given that polar liquids typically exhibit high absorption losses in the THz regime, we evaluated the impact of the imaginary part κ of the refractive index on the spectral response. Referring to the literatures for isopropyl alcohol with $\alpha \approx 50$ cm^{-1}, ethanol with $\alpha \approx 60$ cm^{-1}, methanol with $\alpha \approx 100$ cm^{-1}, and glycerol with $\alpha \approx 110$ cm^{-1} even at high temperature, the corresponding imaginary parts are calculated as $\kappa \approx 0.064$, 0.077, 0.129, and 0.14, respectively, using the relationship of $\kappa = \alpha\lambda/4\pi$. As shown in Figure S3, the top x-axis indicates the absorption coefficients α corresponding to each imaginary part κ. As imaginary part κ increases from 0 to 0.14, the resonance linewidth FWHM broadens from 1 GHz to 27 GHz. This indicates that the resonance can maintain a satisfactory linewidth that is narrower than those of conventional THz sensors for liquid sensing.

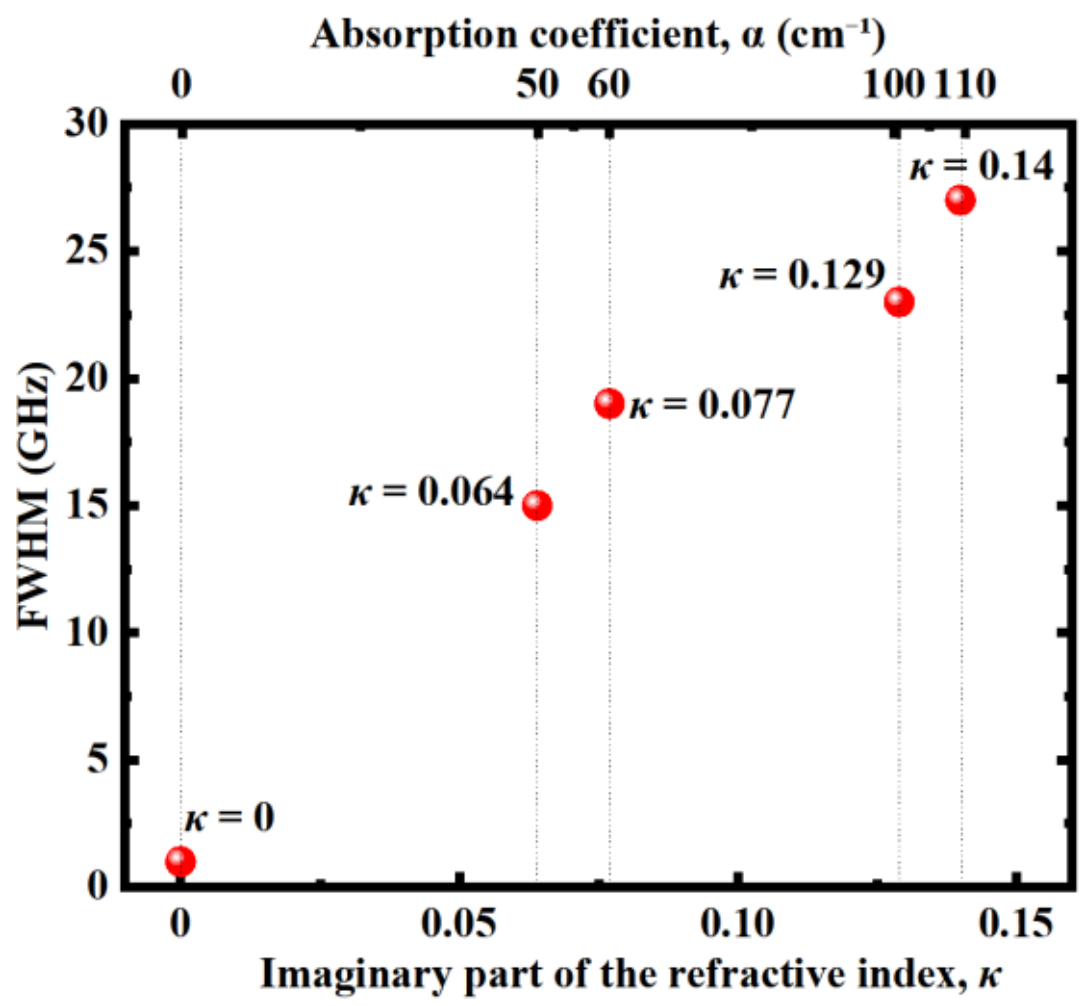

Figure S3. Impact of the imaginary part κ of the refractive index on the resonance linewidth FWHM of the spectral response.

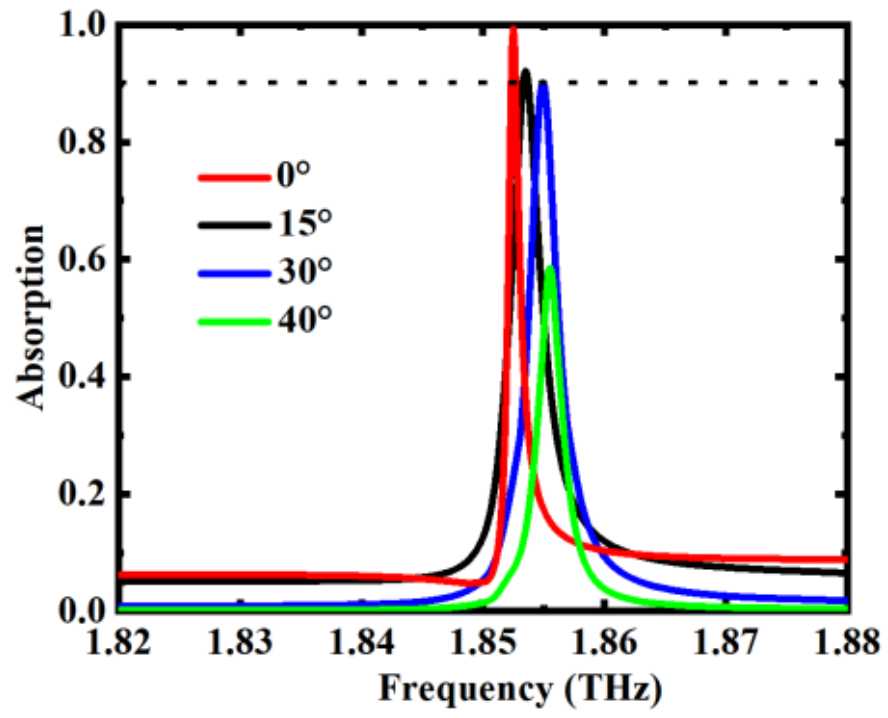

Figure S4. Simulated results of the high-Q Fano resonance under normal incidence and oblique incidence at 15°, 30°, and 40°.

Figure S4 presents the simulated high-Q Fano resonance under normal and oblique incidence at 15 °, 30 °, and 40 °. The resonant absorption maintains high absorbance of over 0.9 for incidence angles up to 30 °, accompanied bya slight blueshift in the resonance frequency. At incidence angles exceeding 40 °, absorption decreases rapidly. The results indicate the device's robustness to oblique incidence up to 30 °.

To systematically unravel the absorption dynamics inherent in this resonant configuration, we analytically derive the effective optical impedance based on the constitutive relation:

$$Z_{eff} = Z'_{eff} + Z''_{eff} \tag{1}$$

The reflection coefficient R is expressed in terms of the optical impedance as:

$$Z_{eff} = \sqrt{\frac{(1+S_{11})^2 - S_{21}^2}{(1-S_{11})^2 - S_{21}^2}} \tag{2}$$

$$R = \left| \frac{Z_{eff} - 1}{Z_{eff} + 1} \right| \tag{3}$$

where S_{11} and S_{21} are the scatteringparameters corresponding to reflection and transmission for normally incident light, respectively. Due to the presence of the gold reflective layer, S_{21} is zero. As quantitatively demonstrated in Fig. S5, the effective optical impedance approaches unity (Z_{eff} = 1) precisely at perfect absorption. This impedance matching condition manifests as the suppression of resonant reflection (R = 0), thereby establishing a direct causal relationship between impedance matching and complete electromagnetic energy dissipation[2].

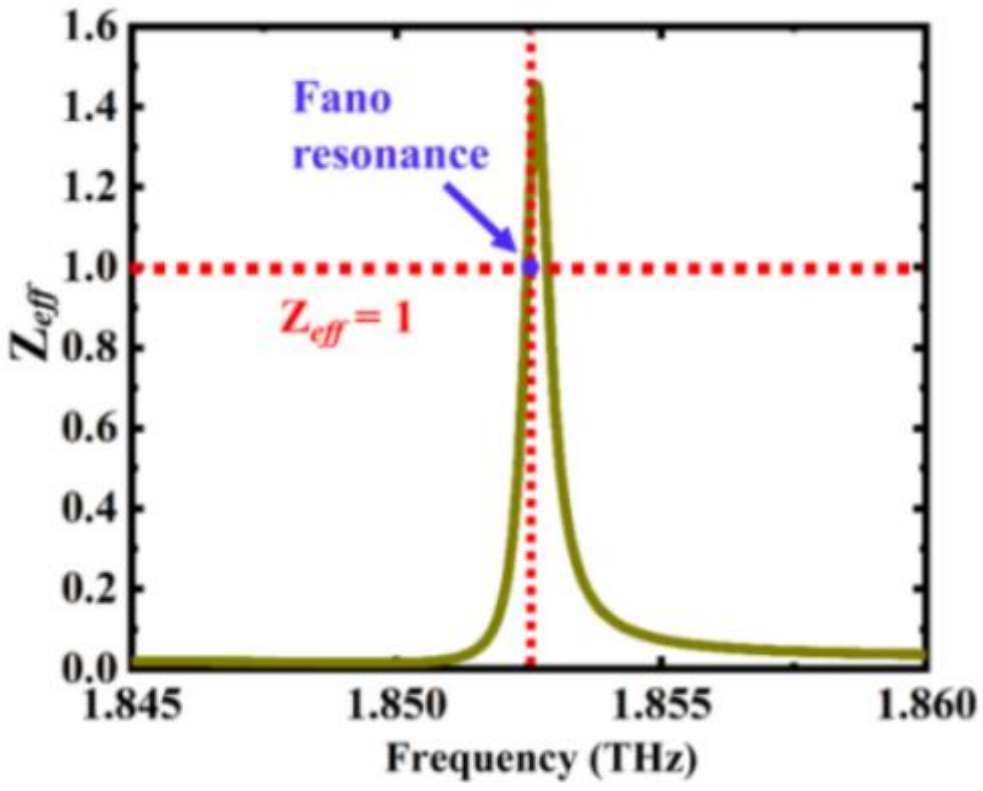

Figure S5. Variation of effective optical impedancenear perfect absorption.The red dashed line

indicates an effective optical impedance of 1 at the resonance frequency.

Fig. S6 shows the variations of absorption and linewidth as a function of gate voltage for three different graphene-strips array arrangements with w = 0.45p, w = 0.5p, and w = 0.55p.

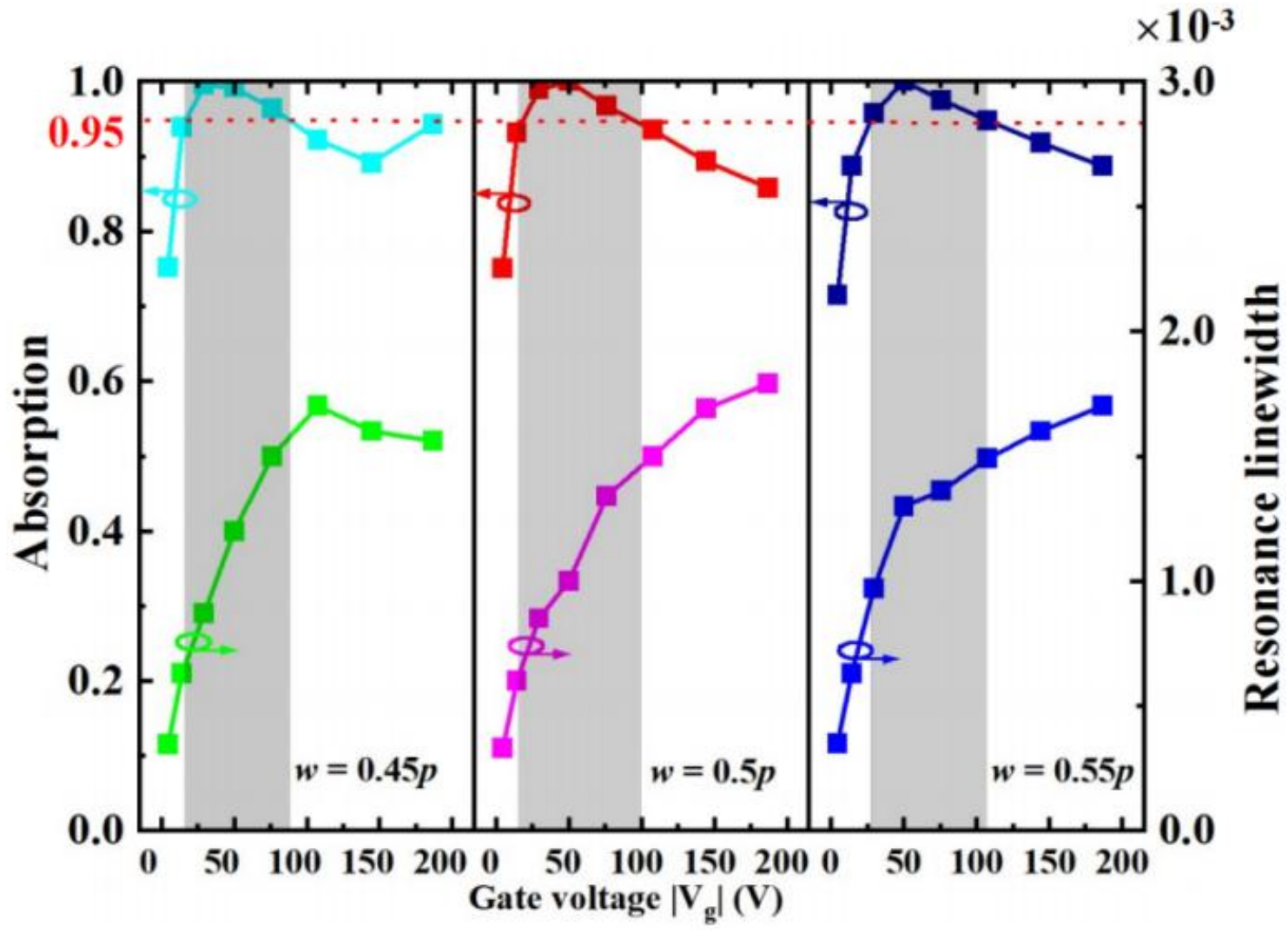

Figure S6. Variations in absorption and linewidth as a function of gate voltage for three different arrangements of graphene-strip arrays. The red horizontal dashed line indicates an absorption level of 0.95. The gray regions denote gate voltage ranges with absorption exceeding 0.95.

The variation of the FOM with cavity length deviation Δl is shown in Figure S7. The FOM attains ~10^6 under optimalconditionsAs shown in Figure S7, a cavity length deviation of ±2 μm reduces the FOM to the order of 10^5. For larger deviations up to ±4 μm, the FOM further decreases, yet a satisfactory level of 10^4 can be still guaranteed. Herein, the blue dashed line indicates the FOM level of 10^4.

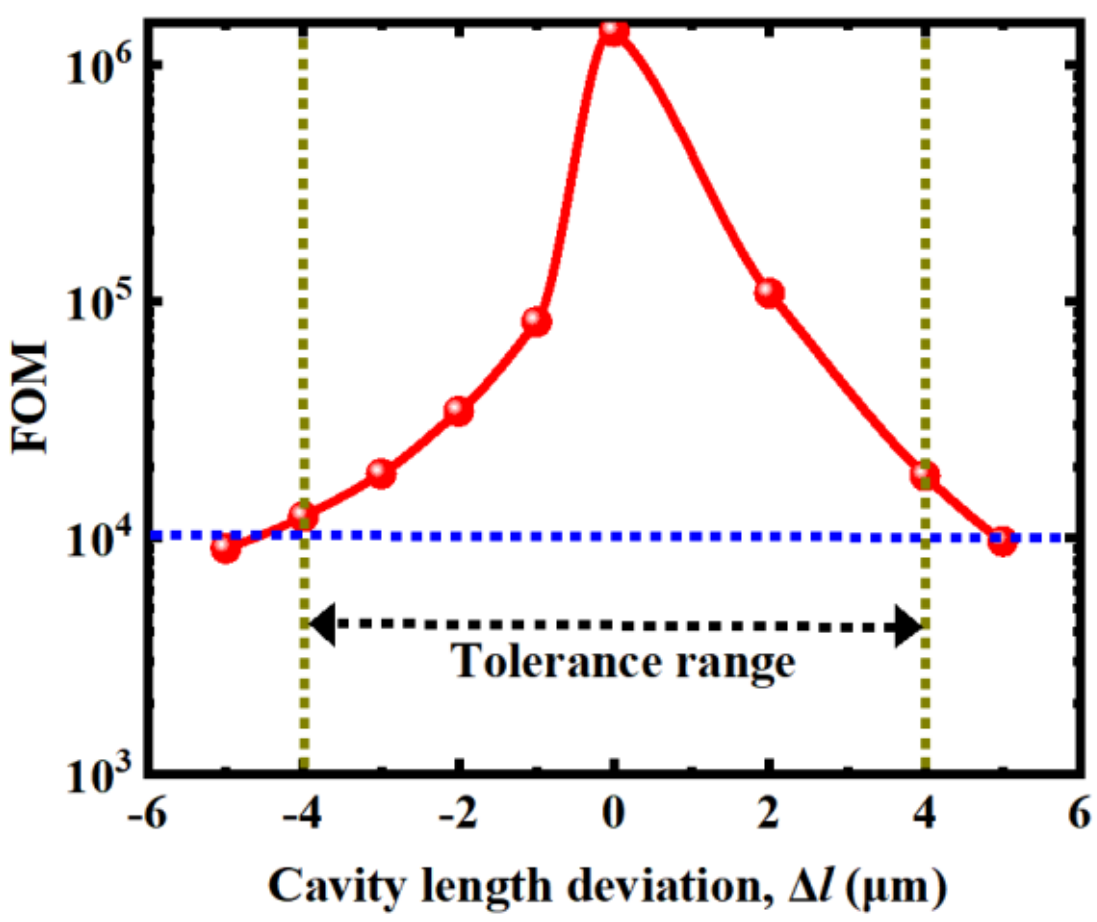

Figure S7. Fabrication tolerance analysis of the FOM.

FOM Tuning with Stable Frequency

The key advantage of graphene lies in the dynamic tunability of its sensing performance, rather than merely in the pursuit of high Q-factors and narrow FWHMs. With highly stable sensitivity maintained, this unique performance can be fully exploited by adjusting the graphene gate voltage

and selecting an appropriate operating range (e.g., where FOM increases). This is particularly beneficial for applications requiring real-time tuning and complex environmental detection. To elucidate the electrical tuning of graphene-strips, the evolution of absorption spectra at specific gate voltages ($|V_g|$ = 14.3 V, 50.1 V, 107.5 V, 186.7 V) is investigated for a cavity thinkness of 41 μm and refrective index of 1.8, as shown in Fig. S8(a). These voltages correspond to Fermi levels of 0.23 eV, 0.43 eV, 0.63 eV to 0.83 eV. The results demonstrate that while the spectral lineshape undergoes significant modification with gate voltage tuning, the resonance frequency remains remarkably stable. Although gate voltage variations alter the carrier concentration and distribution, the resonance frequency of localized surface plasmons is primarily determined by the wave vector rather than the Fermi level, due to linear dispersion relation of graphene.Moreover, the geometric confinement effect of the stripe array endows the resonance mode with robust stability against gate voltage variations. The corresponding resonance linewidth FWHMs and Q-factors are discussed. As the gate voltage $|V_g|$ of graphene changes from 14.3 V to 186.7 V, the FWHM is 1.05×10^{-6}, 1.95×10^{-6}, 2.86×10^{-6} and 6.00×10^{-6}, respectively, indicating progressive linewidth broadending.The increasing linewidth of the Fano resonance with increasing gate voltage can be attributed to the significant rise in carrier concentration. This elevated carrier density intensifies scattering processes, substantially reducing the carrier relaxation time τ. Consequently, the resonance linewidth brodens according to the inverse relation $\Delta\omega \propto 1/\tau$[29-31]. The Q-factor decreases characteristic monotonically, with corresponding values of 1.85×10^{6}, 9.93×10^{5}, 6.78×10^{5}, and 3.23×10^{5}, respectively. Although higher gate voltages reduces the Q-factor (introducting a trade-off), it remains exceptionlly high values ($>10^5$) even at $|Vg|$ = 186.7 V, owing to the robust plasmonic field confinement. Quantitative analysis shows that the Q-factor exhibits orders-of-magnitude tunability (10^5~10^6)via gate voltage modulation. Fig. S8(b) systematically presents the gate voltage-dependent sensitivity characteristics across a wide operational range ($|Vg|$ = 14.3 V ~186.6 V) and for cavity refractive index spanning 1.4 to 1.8. Notably, the resonance mode sensitivity remains remarkably stable (< 1% variation) under substantial gate voltage modulation. This exceptional performance can be attributed to the unique hybrid Fano resonance mechanismat work in this system. Meanwhile, gate voltage modulation of graphene primarily affects the absorption intensity and spectral lineshape via carrier density tuning, with negligible influence (< 0.1% shift) on the resonant frequency. Fig. S8(c) illustrates the dependence of the FOM on gate voltage $|V_g|$ for different refractive index n_c under coherent Fano resonance. At a given gate voltage, as the refractive index n_c of the analytes increases from to1.8, the FOM exhibits a non-monotonic trend of initially decreasing then increasing with the cavity refractive index n_c, primarily due to the synergistic interplay between coherent Fano resonance and the electrically tuning mechanism of graphene.

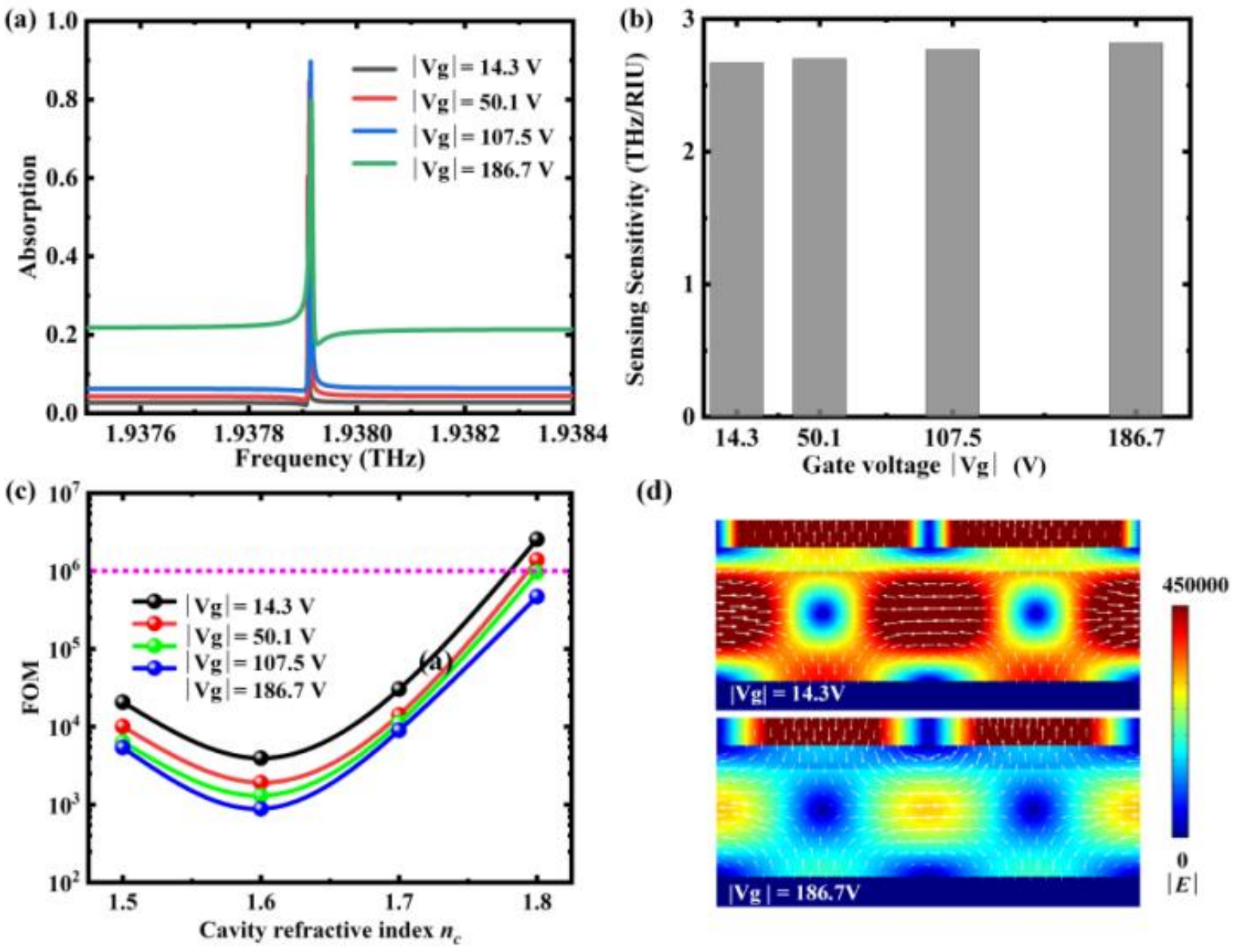

Fig. S8. (a) Evolution of absorption spectra as a function of gate voltage $|V_g|$; (b) Sensitivity variations as a function of gate voltage $|V_g|$; (c) FOM versus gate voltage $|V_g|$ for different cavity refractive index n_c; (d) Electric field intensity distributions under low and high gate voltage, respectively, using the same color scale.

Strong coherent Fano resonance is exceptionally sensitive to small perturbations in the dielectric environment. For a given refractive index, , the FOM exhibits a tuning range of one order of magnitude as the gate voltage gradually increases. At a high refractive index of 1.8 and low gate voltage $|V_g|$ of in the range of 14.3 V ~ 107.5 V, the FOM can reach 10^6—an improvement of at least three orders of magnitude compared to conventional THz graphene sensors[11,20]. Although the absolute FOM value remains high ($>10^3$), suboptimal coupling causes a slight decline in FOM as n_c increases. Near the critical refractive index $n_c \approx 1.6$, the graphene LSPR mode, gold SPP mode, and cavity mode interact, approaching a critical coupling point, leading to temporary FWHM broadening.. This results in a local minimum in the FOM. However, the high electron mobility of graphene maintains low intrinsic losses, keeping the FOM above 10^3 even at higher gate voltage $|V_g|$ of 186.7 V. In the high refractive index range of 1.7~1.8, further increases in n_c significantly alter the dielectric environment between the analyte and the graphene-strips metasurface, thereby enhancing the localized fields of LSPR, SPP, and cavity mode. The resonant frequency shift ($\Delta f/\Delta n_c$) increases, while Fermi level tuning of the graphene-strips metasurface via gate voltage $|V_g|$ compresses the FWHM, ultimately elevating the FOM to 2.54×10^6 at $n_c = 1.8$. This phenomenon can be attributed to the significantly enhanced electric field localization at the low gate voltage of $|V_g| = 14.3$ V, compared to that at the high gate voltage $|V_g| = 186.7$ V, as clearly demonstrated in Fig. S8(d). The proposed structure exhibits remarkable electric field enhancement at three critical regions, including the edges of graphene-strips, the microcavity region, and the gold film surface. Hot spots originating from graphene-diople LSPR with extreme field enhancement can significantly enhance light-matter interaction and boost sensitivity. Standing wave pattern becomes more pronounced in the resonant cavity, leading to improved field confinement and increased Q-factor due to reduced radiation losses. The contributes to an FOM improvement from 10^5 (at high $|V_g|$) to $> 10^6$ (at low $|V_g|$) when the cavity refractive index is 1.8, making the 14.3V operating point ideal for high-sensitivity detection.

Table S1 summarizes the sensing performance of recently reported theoretical THz sensors including sensitivity, normalized sensitivity, and FOM [3-10],with consistentmetric definitions across all schemes. For refractive index sensing, the proposed scheme achieves a high sensitivity of 2.7 THz/RIU and a normalized sensitivity of ~1.5/RIU. Most notably, it attains an exceptional FOM of 2.54×10^6. By comparison, the proposed scheme demonstrates outstanding advantages across all performance metrics for refractive index sensing detection. Although the graphene subwavelength plasmonic grating exhibits significant sensing sensitivity, its normalized sensitivity remains relatively low. In contrast, the proposed approach achieves a normalized sensitivity nearly 3.5 times higher than that of graphene subwavelength plasmonic gratings, while demonstrating a remarkable improvement in FOM by a factor of 2×10^5. Comprehensive evaluation confirms that our proposed scheme maintains high sensitivity while significantly enhancing the FOM, achieving a comprehensive breakthrough in sensing performance. This strategy integrates the sensitivity of optical sensing with the stability and convenience of electrical readout, showing great potential for biomedical and environmental monitoring applications.

Table S1. Comparison of sensing performance among recently proposed advanced theoretical THz sensors

Sensor proposals	Sensitivity [THz/RIU]	Frequency [THz]	Normalized Sensitivity [RIU^{-1}]	Max FOM
Graphene subwavelength plasmonic grating[11]	10.0	25.9	0.39	12.5
Metal cross metamaterial absorber[19]	3.50	6.40	0.55	7
Metal/graphene metamaterial[20]	1.90	11.5	0.17	6.56
Dielectric diffraction grating[26]	0.52	~1	0.53	615
Frequency selective metasurface[54]	0.14	0.70	0.20	0.6
Parallel-plate waveguide resonant cavities[55]	0.22	0.27	0.81	~ 73
Unity integration of grating slot waveguide and microfluid[56]	0.44	0.98	0.45	692
all-dielectric metasurface[57]	0.49	2.2	0.22	2.535×10^4
This work	2.7	~2	~1.5	2.54×10^6
